# Higher-order partial least squares for predicting gene expression levels from chromatin states

**DOI:** 10.1186/s12859-018-2100-y

**Published:** 2018-04-11

**Authors:** Shiquan Sun, Xifang Sun, Yan Zheng

**Affiliations:** 1 School of Computer Science, Northwestern Polytechnical University, Xi’an, 710072 Shaanxi People’s Republic of China; 2 School of Science, Xi’an Shiyou University, Xi’an, 710065 Shaanxi People’s Republic of China; 3 Department of Biostatistics, University of Michigan, Ann Arbor, 48109 MI USA

**Keywords:** Higher-order partial least squares, Chromatin states, Tensor decomposition, Gene expression levels, Histone modification

## Abstract

**Background:**

Extensive studies have shown that gene expression levels are strongly affected by chromatin mark combinations via at least two mechanisms, i.e., activation or repression. But their combinatorial patterns are still unclear. To further understand the relationship between histone modifications and gene expression levels, here in this paper, we introduce a purely geometric higher-order representation, *tensor* (also called multidimensional array), which might borrow more unknown interactions in chromatin states to predicting gene expression levels.

**Results:**

The prediction models were learned from regions around upstream 10k base pairs and downstream 10k base pairs of the transcriptional start sites (TSSs) on three species (i.e., Human, Rhesus Macaque, and Chimpanzee) with five histone modifications (i.e., H3K4me1, H3K4me3, H3K27ac, H3K27me3, and Pol II). Experimental results demonstrate that the proposed method is more powerful to predicting gene expression levels than several other popular methods. Specifically, our method enable to get more powerful performance on both commonly used criteria, R and RMSE, as high as 1.7% and 11%, respectively.

**Conclusions:**

The overall aim of this work is to show that the higher-order representation is able to include more unknown interaction information between histone modifications across different species.

## Background

In epigenetics, histone modifications like methylation, acetylation, and phosphorylation play critical roles in transcriptional regulation process [1]. Specifically, during gene expression process, each unit of chromatin like beads wrapping around DNA subsequences (about 147 base pairs) is highly impact the process of gene expression by chemical modification of chromatin condensation and DNA accessibility when genetic information are converted into gene products [2]. These modifications are shown to regulate gene transcription with active or repressive manners [3]. For example, tri-methylation on K4 of histone H3 (i.e., H3K4me3) is primarily associated with transcriptional activation [4, 5], while tri-methylation on K27 of histone H3 (i.e., H3K27me3) are primarily associated with transcriptional repression [6, 7].

One of challenges in this study is to discover or characterize what chromatin mark combinatorial patterns can affect the process of gene expression, further revealing complex gene expression mechanisms in downstream analysis [8–13]. This topic have attracted extensive attentions [14–16], however, up to now it is still limited knowledge to understand the degree of complexity of “histone code”. Recent studies have shown that machine learning-based methods can statistically offer higher prediction power to predict gene expression levels, and it can be considered as a promising way to reveal some interesting results in many cases ([17, 18], Devadas L, Yen A, Kellis M. Various localized epigenetic marks.predict expression across 54 samples and reveal underlying chromatin state enrichments. 2015; bioRxiv 030478, unpublished). For example, Chen et al., utilized support vector machine to train a prediction model for each bin. The results demonstrated that all bins are useful to predict gene expression levels, but they are not equally informative. In order to investigate the higher-order interactive relationship between chromatin features, they modeled an interaction model $ y \sim {\sum \nolimits }_{i} {{x_{i}}} + {\sum \nolimits }_{i < j} {{x_{i}}{x_{j}}} $ to predict gene expression levels, where the expression level *y* as a linear combinations of the interactions between individual histone modification features *x*_*i*_ and their products *x*_*i*_*x*_*j*_ [19]; Dong et al., established a two-step model using linear regression model and random forest method to reveal the relationship between chromatin features and gene expression levels across various cellular contexts. The best bin was selected to represent the remaining signals for each histone modification. The predictor matrix was formed from the best bin for each histone and the whole gene expression levels [20]; Zhou et al., developed a linear mixed model to evaluate the association of each and joint contribution of the five marks with gene expression levels. The marginal effects of each mark are the summation of all window size. The higher-order interactions between markers were also studied by considering them as the covariates in linear mixed model [21].

To naturally characterize higher-order interactions between different markers, tensor representation (also called multilinear or *N*-way) are frequently introduced to model higher-order interactions in different research fields [22, 23]. More recent studies ([24], Khan SA, Ammad-ud-din M. tensorBF: an R package for Bayesian tensor factorization. 2016; bioRxiv 097048, unpublished) leveraged tensor representation to integrate different omics, environmental, and phenotypic data sets to uncover unclear biological problems; Also, our previous work [25] used tensor representation to identify transcription factor binding sites. All results from these applications are demonstrated that tensor representation enable to achieve a powerful performance.

**Fig. 1:**
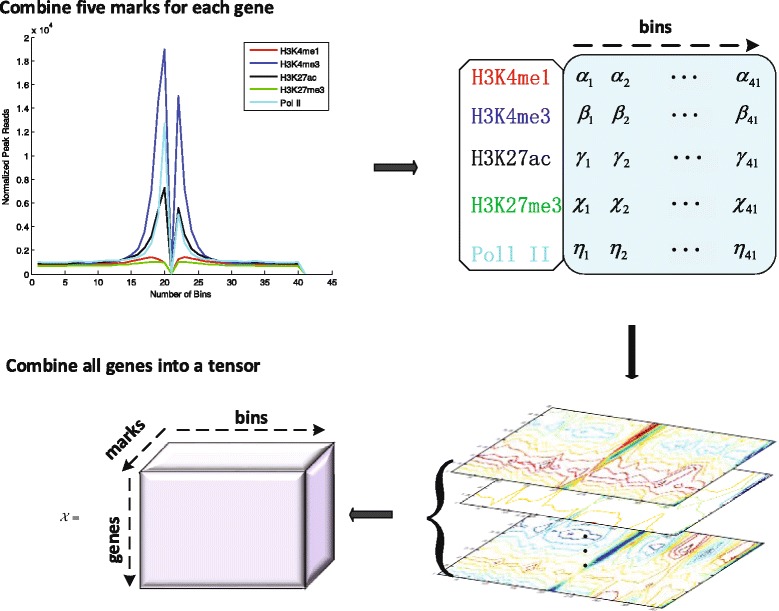
The process of constructing the tensor data ${\mathcal {X}} $ from the peak reads enrichments near TSSs

In this paper, we leverage tensor representation, which intuitively involves more interaction information for chromatin features, to predict gene expression levels. The predictors for each gene were represented by a matrix as input(rather than a vector), in which each row indicates histone markers while each column represents the bin we combined (see Fig. 1). To make the proposed method scalable, three popular machine learning-based methods, including linear regression, random forest [26] and support vector regression [27], were conducted on a series of simulation and real data sets. The results demonstrate that the proposed method gave a statistically significant improvement compared with other prediction models.

## Methods

### Data sets and pre-processing

In this study, we used the real data sets which are from lymphoblastoid cell lines (LCLs) over three species, namely Human (GSE47991 and GSE19480), Rhesus Macaque (GSE60269), and Chimpanzee (GSE60269), and these data set are all available in Gene Expression Omnibus (GEO). For each species, eight individuals were considered, and for each individual, 26,115 genes were considered in our experiments.

The preprocessing workflow of real data was completely consistent with the previous work [21]. Five histone marks were queried to contribution in gene expression levels: promoter marks (H3K4me1, H3K4me3, H3K27ac), repressor mark (H3K27me3), and Pol II mark. The reason we choose these five marks not only because their molecular functions have been relatively well studied, but also because they represent a wide variety of transcription initiation regulators. H3K4me1 mark presents at both active and poised enhancers; H3K4me3 mark actives transcription start sites; H3K27ac mark actives enhancers and promoters; H3K27me3 mark represses genomic regions; Pol II directly interacts with chromatin remodeling factors and catalyzes the transcription of mRNA. The actual gene expression levels are measured by RNAseq and quantified as RPKM (reads per kilobases per million mapped reads). We also normalized the real data set with two steps for each species:

(i) We used COVERAGEBED tool [28] to convert the reads into the given window for each mark and each individual, and then we normalized the peak read counts for each individual of each mark by subtracting the number of mapped reads divided by total number of mapped reads and input reads divided by total number of input reads (the detailed procedure see the reference [21]);
(ii) We used logarithmic transformation log2(*x*+*θ*) to normalize the data. In order to obtain the optimal parameter *θ* in prediction phrase, we divided the whole data set into two parts. One-third of data set was used to optimize the parameters *θ*, then the optimized *θ*^∗^ was added to the same modification of the remaining two-thirds of data set to train the prediction model and test their performance.

### High-order representation

In this section, we give more detailed description how the original data were represented by a higher-order representation. The first step is to divide the each gene body, which flanking TSSs both sides with 10k base pairs, into different bins for each individual and each mark (the first two steps of Fig. 1). For each gene, the data for each bin and each histone marker was reformulated into a three-dimension data structure (genes × marks × bins), therefore, each gene was represented by a matrix instead of a vector. The gene expression levels were used the averaged values across 8 individuals. The detailed process to form tensor data is showed in Fig. 1.

As shown in Fig. 1, the first step is to show the different signal patterns near the TSSs over different marks. The histone marks H3K4me3 and Pol II show more informative, while H3K27me3 and H3K3me1 show weaker informative. Each mark was represented by multiple bins (e.g., 41 bins). Therefore, we first combined the five marks into a matrix for each gene. In third step (Fig. 1), we used the contour of distribution of each gene to represent its signals. Assume we have 26115 genes. Finally, we collected all genes to form a tensor data $\mathcal {X}$.

### High-order model and algorithm

Higher-order partial least squares (or *N*-way partial least squares, NPLS) was proposed by Bro et al. [29]. It is adapted to high-order data . Here, *N* is the number of order for high-order data ${\mathcal {X}}$ (in our case, *N*=3), and the variable *I*_*i*_ represents the dimensionality of the mode *i*. The response variable  is the averaged gene expression levels across eight individuals for all marks.

The optimization model of NPLS is easily reformulated from standard PLS model as:

1 $$\begin{array}{*{20}l} \underset{\{\mathbf{P^{(n)}}\},\mathbf{q}}{\max} & \left[{\text{cov}} \left({\mathcal{X}\times_{(2)}\mathbf{P}^{(1)T}\times_{(3)} \!\cdots\! \times_{(N)}\mathbf{P}^{(N-1)T},\mathbf{Y}\mathbf{q}} \right)\right]^{2} \end{array} $$

2 $$\begin{array}{*{20}l} \quad \text{s.t.} &\quad {\mathbf{P}^{(n)T}} \mathbf{P}^{(n)} = \mathbf{I},\,{\mathbf{q}^{T}}\mathbf{q} = 1. \end{array} $$

To solve this model, we want to find the optimal **p**_1_ and **p**_2_ such that:

$${\mathcal{X}} = {\mathbf{t}_{1}} \otimes {\mathbf{p}_{1}} \otimes {\mathbf{p}_{2}} + {{\mathcal{E}}_{1}}$$

where the operation ⊗ is the outer product. The first latent variable  is extracted from the tensor data $\mathcal {X}$ to provide the maximum of covariance between **t**_1_ and the response variable **Y**. The  and  is the loading vector for mode 2 and 3, respectively and ${\mathcal {E}}$ is the residual of data $\mathcal {X}$ after the first extraction. For the given number of factors *f*, the predicted variable can be estimated by the equation,

$$\hat{\mathbf{Y}} = \mathbf{T}\mathbf{b}$$

where **T**=(**t**_1_,**t**_2_,⋯**t**_*f*_), **b** is the regression coefficient with respect to **T**. The MATLAB code of *N*-way partial least squares is freely available at: http://www.models.life.ku.dk/source/nwaytoolbox.

**Fig. 2:**
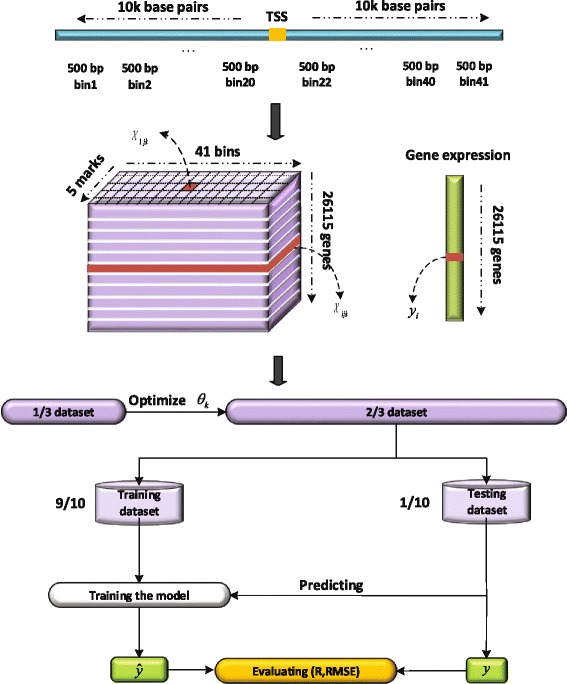
Schematic diagram of the process of data analysis in our paper

The workflow of our experiments was given in Fig. 2. We first extended upstream region and downstream region to 10k base pairs around TSSs, and then divided into multiple bins (e.g., 41 bins if 500 base pairs for each bin). Finally, gene expression levels were measured by a 3-order tensor rather than a matrix, i.e., 26115 genes ×5 marks ×41 bins. The histone density in each bin was logarithm-transformed by log2(*x*+*θ*_*k*_) with respect to the parameter *θ*_*k*_ (the parameter for *k*th bin). In order to determine the optimal $\theta ^{*}_{k}$ for each bin, we divided the whole data into two parts: one-third of dataset was used for finding the optimal parameter $\theta ^{*}_{k}$ and then the same $\theta ^{*}_{k}$ was added to the corresponding bins in the remaining data set. The gene expression levels **Y** was also logarithm-scaled using the equation log2(**Y**). A high-order multivariate regression model was developed using the logarithm-scaled training data set and the 10-fold cross validations was used to avoid the over-fitting in training model phrase. Finally, the performance of methods were measured by the Pearson’s correlation coefficient (R) and root mean square error (RMSE).

## Results

In our experiments, to avoid the risk of over-fitting to training prediction model and obtain the reliable results, we used 10-fold cross-validation (with 10 random splitting replicates) in which nine parts are used for training the prediction model, while the remaining part for testing the performance of learned model (similar to previous study [30]).

We also compared with other three popular machine learning-based methods, i.e., simple linear regression (denoted as LR), random forest (denoted as RF), and support vector machine regression (denoted as SVR) to make the proposed method scalable. All these methods were implemented in R, and the parameter of random forest (the number of trees) was set as 500, and support vector regression used default parameters in R. The performance of each methods were evaluated by two criteria, namely Pearson correlation coefficient (R) and root mean square error (RMSE) which are formulated by the following equations:

$$R = \frac{{{\sum\nolimits}_{i = 1}^{I_{1}} {\left({{\mathbf{Y}_{i}} - {\mu_{\mathbf{Y}}}} \right)\left({{{\hat{\mathbf{Y}}}_{i}} - {\mu_{\hat{\mathbf{Y}}}}} \right)} }}{{\sqrt {{\sum\nolimits}_{i = 1}^{I_{1}} {{{\left({{\mathbf{Y}_{i}} - {\mu_{\mathbf{Y}}}} \right)}^{2}}}} \sqrt {{\sum\nolimits}_{i = 1}^{I_{1}} {{{\left({{{ \hat{\mathbf{Y}}}_{i}} - {\mu_{\hat{\mathbf{Y}}}}} \right)}^{2}}}} }}$$

$$RMSE = \sqrt {\frac{{{\sum\nolimits}_{i} {{{\left({{\mathbf{Y}_{i}} - {{ \hat{\mathbf{Y}}}_{i}}} \right)}^{2}}} }}{I_{1}}} $$

where **Y** is the real gene expression levels, while ${ \hat {\mathbf {Y}}}$ is the predicted the expression levels; *I*_1_ is the number of genes; *μ*_**Y**_ is the mean of gene expression levels **Y**.

### Experiments on simulation data sets

Our first experiments were conducted on a series of simulation data sets. Interestingly, we found that the distribution of each mark is similar to the normal distribution but does not exactly the same as it. Therefore, in this simulation experiments, we simulated the five different histone marks according to their expression levels, which are described the height *h* of their distributions, *h*=3,2,1,0.5,0.4. 10,000 genes were simulated and the distributions of expression levels for each gene were divided into 100 bins to represent the expression levels of corresponding simulated histone. Similar to the PLS model, we also introduced a latent variable *Z* to simulate the data in simulation experiments.

Suppose the latent variable *Z*∼*N*(0,*β*), then

$$y = Z + {\varepsilon^{y}} \quad \text{ and} \log \left({\sigma_{i}^{2}} \right) = Z \times {h_{i}} + \varepsilon_{i}^{\sigma} $$

where *ε*^*y*^∼*N*(0,0.2) and $\varepsilon _{i}^{\sigma } \sim N\left ({0,{\gamma _{i}}} \right), i=1,2,3,4,5$.

The simulation data $\mathcal {X}$ with 100 bins can be obtained by partitioning the density function $N \left ({0,\sigma _{i}^{2}} \right)$ into 100 intervals and calculating their area of corresponding intervals for each mark.

**Fig. 3:**
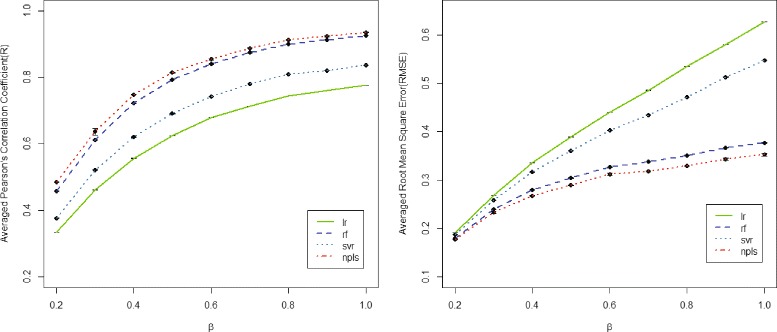
Comparison of four algorithms for predicting gene expression level. The left figure demonstrates the averaged correlation coefficients with varies parameter *β* over 10 random splitting replicates while the right figure shows the averaged root mean square error with varies parameters *β* over 10 replicates. lr: linear regression; rf: random forest; svr: support vector regression; npls: *N*-way partial least squares

Figure 3 shows the prediction of four methods with respect to varies parameters *β*. As shown in Fig. 3, our method (npls) steadily outperforms others on both criteria, i.e., R (left) and RMSE (right). The second-best performance is achieved by random forest regression. Compared with other three methods, linear regression method linearly increases on RMSE.

### Experiments on real data sets

Our second experiments were conducted on real data sets. In this section, we investigated the relationship between gene expression level and chromatin features based on three species (Humans, Chimpanzees, and Rhesus Macaques). The results from the previous work [21] have shown that five marks are significantly enrich near TSSs regardless of species, and the enrichments pattern is robust with respect to the choice of the size of the TSS regions.

**Fig. 4:**
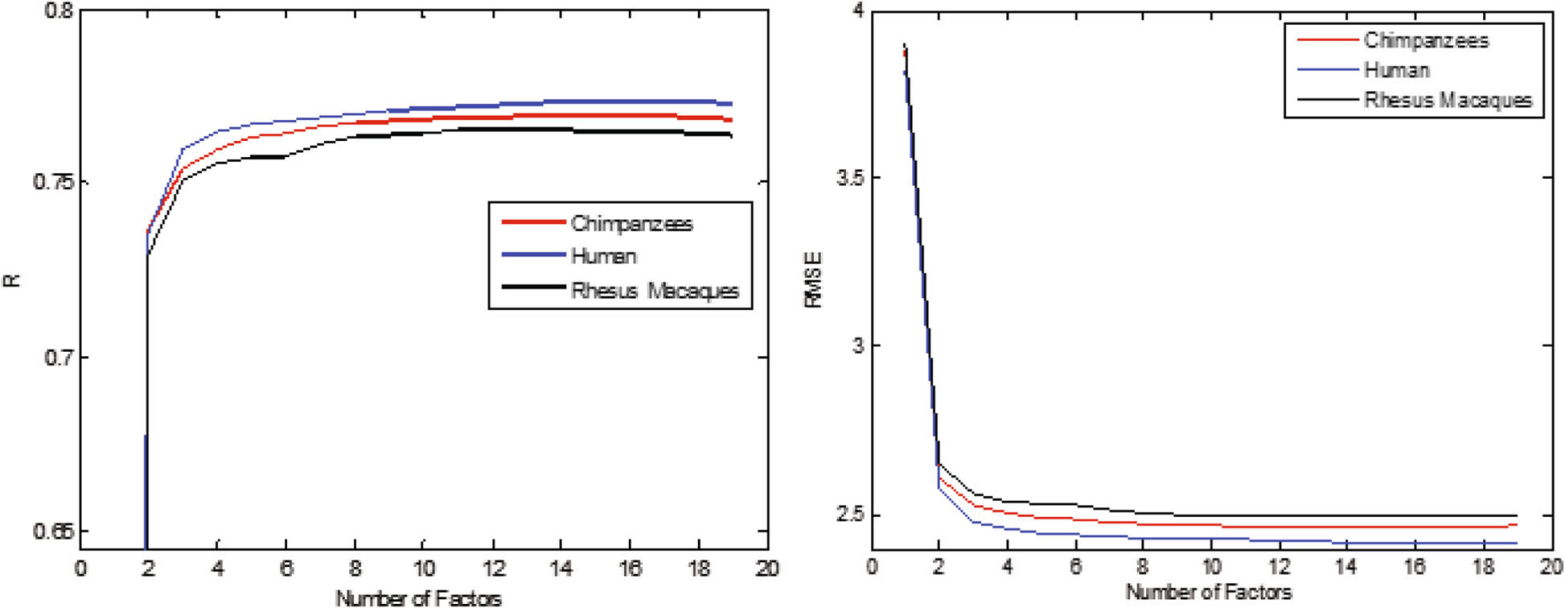
The relationship between the numbers of factor and the performance on three species

Herein, we considered the DNA regions around (10k) at the upstream and downstream regions of the TSSs in current study. In our model, the number of factors is an important parameter to affect the performance. To investigate how this parameter affect the performance of proposed method, we check it under two criteria (R and RMSE) with the varies of factors (see Fig. 4). We can see that the performances are robust when the number of factors is larger than 4.

The comparison of the results of four regression models over three species were summarized in Table 1. As shown in Table 1, our method is steadily better than others with respect to both averaged R and averaged RMSE on three species. For the performance on R, the proposed method improved roughly 1.2%, 1.7%, and 1.3% on Hum, Chi, and Rhe data sets, respectively, while RMSE was improved roughly 11%, 8%, and 8% on Hum, Chi, and Rhe data sets, respectively. For other methods, random forest regression outperforms other two methods on Hum and Chi data sets while support vector machine outperforms other two methods on Rhe data set.

**Table 1:** The performance of different models on three species data sets

	Linear model	Random forest	Support vector machine	NPLS(41bins)	NPLS(21bins)
Hum	0.769(2.43)	0.775(2.46)	0.774(2.46)	0.784(2.37)	0.787(2.35)
Chi	0.756(2.52)	0.767(2.47)	0.765(2.53)	0.780(2.41)	0.784(2.39)
Rhe	0.760(2.52)	0.761(2.51)	0.765(2.54)	0.774(2.46)	0.778(2.43)

Note: The number in bracket following the average R represents averaged RMSE over 10-flod cross validation (with 10 random splitting replicates). Hum: Human data set, Chi: Chimpanzee data set, and Rhe: Rhesus Macaque data set

## Discussion and conclusion

In this paper, we proposed a higher-order representation method for predicting gene expression levels from chromatin state enrichments. The effectiveness of proposed method was validated by a series of simulation and real data sets. Our method can outperforms others, most likely because higher-order representation method can integrate more unknown interaction information than standard representation method. These results again demonstrate that the gene expression levels are strongly correlated with the combination of chromatin markers.

Each chromatin state shows specific functional and annotation. Our study provides a way to study genomic annotation via chromatin mark combinations, which can extend the epigenetic functional interpretation of the human genome. Therefore, our further work is to incorporate epigenetic factors into the downstream analysis, such as gene expression analysis [9, 31], GO ontologies [32, 33], and disease-related ncRNAs [34].
